# Changes in root chemical diversity along an elevation gradient of Changbai Mountain, China

**DOI:** 10.3389/fpls.2022.897838

**Published:** 2022-11-07

**Authors:** Shihua Wu, Ruili Wang, Haihua Zhu, Yuan Wang, Yanyan Du, Sihao Zhu, Ning Zhao

**Affiliations:** ^1^ State Key Laboratory of Grassland Agroecosystems, College of Pastoral Agriculture Science and Technology, Lanzhou University, Lanzhou, China; ^2^ College of Forestry, Northwest A&F University, Yangling, China

**Keywords:** chemical diversity, chemical assembly, belowground, root chemical traits, multi-element, elevation pattern

## Abstract

Root chemical traits play a critical role in plant resource use strategies and ecosystem nutrient cycling; however, the chemical diversity of multiple elements of fine root and community chemical assembly belowground are poorly understood. Here, we measured 13 elements (C, N, K, Ca, Mg, S, P, Al, Fe, Na, Mn, Zn, and Cu) in the fine roots of 204 plant species along elevational transect from 540 to 2357 m of Changbai Mountain, China to explore the variation, diversity, and community assembly of root chemical traits. At the species level, the concentrations of macronutrients (N, K, Ca, Mg, S, and P) decreased, whereas the trace metals (Fe, Mn, and Zn) increased with elevation. Root chemical traits at the community level systematically shifted along elevational gradients showing a pattern similar to that at the species level, which were mainly influenced by climate and soil rather than species diversity. In general, the interactions of climate and soil were the main drivers of root chemical assembly for woody layers, whereas soil factors played significant role for root chemical assembly for herb layer. The chemical assembly of rock-derived element P was mainly driven by soil factors. Meanwhile, root chemical diversities were mainly regulated by species diversity, the interactions of climate and soil, and soil factors in the tree, shrub, and herb layers, respectively. A better understanding of plant root chemical diversity and community chemical assembly will help to reveal the role of chemical traits in ecosystem functioning.

## Introduction

Fine roots play an important role in plant functions and ecosystem processes (Norby and Jackson, 2000; Weemstra et al., 2017; Wang et al., 2021). Fine roots are the primary organs for plant resource acquisition, and nutrient release from decomposing roots is a key pathway for nutrient cycling in terrestrial ecosystems (Yuan and Chen, 2010; Haichar et al., 2014; Sun et al., 2021). Many studies have focused on fine root structure and function, including the architectural structure and root morphology (Pregitzer et al., 2002; Iversen, 2014), root production (Yuan and Chen, 2012) and decomposition (Silver and Miya, 2001; Zhang and Wang, 2015), root economics spectrum (Kong et al., 2014; Zhou et al., 2018; Kong et al., 2019; Han and Zhu, 2021), geographic patterns of root traits (Yuan et al., 2011; De La Riva et al., 2018; Weemstra et al., 2021), coordinated variation in leaf and root traits (Newman and Hart, 2006; Zhao et al., 2016a; Weigelt et al., 2021), and root dynamics under global change (Norby and Jackson, 2000; Wang et al., 2015). Furthermore, the Global Root Trait (GRooT) database was created by integrating widespread root traits to improve our understanding of root functioning (Guerrero-Ramirez et al., 2021). Although research on root traits is developing rapidly, understanding root chemical traits are limited to nitrogen (N) and phosphorus (P). The chemical diversity of multiple elements in fine roots and community chemical assembly belowground are still in their infancy.

Although nitrogen (N) and phosphorus (P) are recognized globally as critical to plant functioning, many other elements are also important in plant growth and community composition (Duval et al., 2013). For example, Carbon, which makes up about 38% of plant dry matter, is the basis of the organic skeleton (Stockinger et al., 1997). Iron (Fe), a component of chlorophyll and many enzymes, is involved in the formation of chloroplast proteins and plays an important role in cellular respiration and metabolism (Koseoglu, 1995). Sulfur (S) is a major component of proteins, amino acids, and enzymes and is involved in promoting REDOX, which participates in chlorophyll formation and carbohydrate metabolism (Wang et al., 2009). Aluminum (Al) is a metallic element that attracts plant growth-promoting rhizobia and its interactions with plants by increasing organic secretions (Muhammad et al., 2019). Potassium (K) and sodium (Na) are closely related to plant photosynthesis and respiration and play important roles in enzyme activation, osmotic regulation, and stomatal opening (Wang et al., 2004). Magnesium (Mg) and calcium (Ca) are important intracellular divalent cations and cofactors in various enzymatic reactions in energy metabolism and protein and nucleic acid synthesis (Fan et al., 2020). Studies have also suggested that K and Ca play a key role in plant adaptation to water stress, for example, plants increase K^+^ and Ca^2+^ uptake to improve drought resistance (Sardans and Peñuelas, 2015; Tian et al., 2019). Metal elements, such as copper (Cu), manganese (Mn), and zinc (Zn), are mainly involved in electron transport during plant growth, acting as enzyme activation centers, and are also components of a variety of secondary metabolites (Kooijman, 1995; Penuelas et al., 2008; Agren and Weih, 2012). Therefore, understanding the variations in root chemical traits is the foundation for elucidating the formation of community chemical diversity.

The variation and driving factors of root chemical traits at the community level are poorly documented relative to our understanding of root chemical traits at individual and species levels. Previous studies have focused on the variation and driving factors of species chemical traits along environmental gradients. Plant functional type (PFT), climate, and soil are considered to be the major factors shaping the biogeographic patterns of root chemical traits in a complex manner. Root N, P, and multiple elements differed significantly among different biomes, vegetation types, and PFT (e.g., root P increased from tropical forests, tropical grasslands, temperate forests to temperate grasslands, and boreal forests), favoring the species composition hypothesis derived from leaf chemical geography (Reich and Oleksyn, 2004). Trees, shrubs, and herbs indicate similar latitudinal and elevational trends of root chemical traits, that is, root N:P declined with latitude (Yuan et al., 2011) and increased with altitude (Zhao et al., 2016b). Temperature and precipitation are the two most critical climatic variables that strongly govern vegetation distribution and structure, shaping the spatial patterns of root chemical traits, both directly and indirectly (Yuan et al., 2011). Soil chemical attributes (e.g., pH and mineral nutrient availability) are critical for plant growth, and their impact on root chemical patterns cannot be ignored. Several environment-associated hypotheses have been proposed to delineate the formation of leaf chemical patterns, such as the temperature-biogeochemistry hypothesis, temperature-plant physiology hypothesis, and soil substrate age hypothesis (Reich and Oleksyn, 2004), which are also useful for understanding root chemical patterns. However, these species-level studies are inadequate for accounting for the variations in root chemical traits at community level, which are affected by the abundance of different species within a community and community structure. Therefore, to better understand community chemical diversity and chemical assembly belowground, it is necessary to upscale root chemical traits from species to community level.

Although roots play important roles in community assembly (Laughlin, 2014), our understanding of community chemical diversity and assembly is largely based on aboveground traits (Asner et al., 2014; Diaz et al., 2016; De La Riva et al., 2018; Agren and Weih, 2020; Fernández-Martínez, 2022). Chemical diversity is considered as an important dimension of functional diversity and has been proven useful for understanding ecological niches (Penuelas et al., 2019; Sardans et al., 2021) and ecosystem functioning (Craven et al., 2018; Fernandez-Martinez et al., 2021). Although the relationship between functional diversity and species richness showed discordance in different studies and could vary across spatial scales (Suárez-Castro et al., 2022), studies on chemical traits have indicated increased chemical diversity with coexistence between species and higher species diversity (Fernandez-Martinez et al., 2021). The increased chemical diversity could be explained by the complementarity effect (Cardinale et al., 2007), based on different species in a community with contrasting ecological niches and dissimilar chemical traits. Environmental factors (climate and soil factors) affect the distribution of chemical traits and influence the community’s chemical assembly. The study of forest canopies explored the strong environmental control of canopy chemical assembly, especially for rock-derived elements (Asner et al., 2014). It has also been claimed that environmental filtering is the main driver of root chemical assembly along environmental gradients, particularly climate and soil filters (Zhao et al., 2018a). However, there are few studies on root chemical traits at the community level based on root traits, especially the variation and driving factors of community chemical diversity and assembly for different forest stratifications (tree, shrub, and herb layers). Based on the limited observations above, we hypothesize that the controlling factors (climates, soil properties, and species diversity) of community root chemical diversity and assembly are different among the PFTs. In general, the interactions of climate and soil are the main drivers of root chemical assembly for woody layers, whereas soil factors play significant role for root chemical assembly for herb layer. The chemical assembly of rock-derived element P is mainly driven by soil factors.

The substantial variation in microhabitat structure along an elevational gradient provides an excellent natural laboratory for investigating the responses of root chemical traits to environmental changes. In this study, we investigated the concentrations of 13 elements of fine roots along an elevational transect on the northern slope of Changbai Mountain, China. To test our hypothesis, we analyzed (1) the elevational patterns of root chemical traits at the species level and their controlling factors (climates, soil properties, and PFT), (2) the elevational patterns of root chemical traits at the community level for different PFTs (tree, shrub, and herb layers) and the key controlling factors (climates, soil properties, and species diversity), and (3) the elevational patterns of community root chemical diversity for different PFTs and their controlling factors (climates, soil properties, and species diversity).

## Materials and methods

### Study sites

Changbai Mountain (N41°23′-42°36′, E126°55′-129°00′ E) is located in the northeast of Jilin Province, China and is characterized by a temperate continental monsoon mountain climate. Along the increasing elevational gradient from 500 to 2357 m, mean annual temperature (MAT) decreases from 3.5°C to -7.4°C, and mean annual precipitation (MAP) increases from 700 to 1400 mm (Zhu et al., 2010). There is noticeable vertical climate change and distinct vertical vegetation zonation. As elevation increases, vegetation types range from broad-leaved forest (BL), mixed coniferous broad-leaved forest (MCB), dark-coniferous spruce-fir forests (DCFs), dark-coniferous spruce forests (DCS), Erman’s birch forest (EB) to alpine tundra (AT). The soil types and the main characteristics along the elevational gradient are summarized in **Table 1**.

**Table 1:** Summary of vegetation types, environmental factors, plot numbers, dominant species, and the sample numbers for 204 species from six sites along the elevational transect on Changbai Mountain.

Forest type	Broad-leaved forest (BL)	Mixed coniferous broad- leaved forest (MCB)	Dark-coniferous spruce-fir forest (DCF)	Dark-coniferous spruce forest (DCS)	Ermans birch forest (EB)	Alpine tundra (AT)
Site	Site A	Site B	Site C	Site D	Site E	Site F
Elevation (m)	540	753	1286	1812	2008	2357
MAT (°C)	2.9	2.6	0.3	-2.3	-3.3	-4.8
MAP (mm)	632	691	811	967	1038	1154
STN (mg g^-1^)	7.39	4.92	0.78	3.79	2.71	2.2
STP (mg g^-1^)	1.54	1.36	0.42	0.93	0.51	0.4
SAN (mg kg^-1^)	81.65	70.73	40.81	70.7	61.08	49.67
SAP (mg kg^-1^)	18.18	8.22	17.7	9.2	5.46	7.07
pH	5.31	5.01	5.23	5.03	5.02	5.14
Tree plot number	4	4	4	4	4	4
Shrub plot number	20	16	12	17	24	18
Herb plot number	16	16	16	16	16	16
Tree species number	5	24	12	8	2	0
unique species	1	15	5	3	0	0
Dominant tree species	*Fraxinus mandshurica* Rupr., *Juglans regia* Linn.	*Fraxinus mandshurica* Rupr., *Pinus koraiensis* Siebold et Zuccarini., *Quercus mongolica* Fischer ex Ledebour.	*Pinus koraiensis* Siebold et Zuccarini., *Picea jezoensis* Carr. var. microsperma (Lindl.) Cheng et L. K. Fu, *Larix olgensis* Henry.	*Larix olgensis* Henry., *Larix gmelinii* (Ruprecht) Kuzeneva., *Picea jezoensis* Carr. var. microsperma (Lindl.) Cheng et L. K. Fu.	*Betula ermanii* Cham.	
Shrub species number	22	15	11	8	8	2
unique species	11	8	6	4	5	1
Dominant shrub species	*Philadelphus incanus* Koehne, *Euonymus alatus* (Thunb.) Sieb., *Spiraea chamaedryfolia* Linn., *Corylus mandshurica* Maxim.	*Philadelphus incanus* Koehne, *Deutzia scabra* Thunb.	*Lonicera caerulea* Linn. var. edulis Turcz. ex Herd., *Lonicera praeflorens* Batal.	*Ribes burejense* Fr. Schmidt., *Lonicera caerulea* Linn. var. edulis Turcz. ex Herd.	*Rhododendron aureum* Georgi	*Vaccinium uliginosum* Linn., *Rhododendron redowskianum* Maxim.
Herb species number	43	36	14	28	19	8
unique species	29	22	6	16	13	8
Dominant herb species	*Meehania urticifolia* (Miq.) Makino, *Athyrium sinense* Rupr., *Aegopodium alpestre* Ledeb.	*Cardamine leucantha* (Tausch) O. E. Schulz, *Meehania urticifolia* (Miq.) Makino, *Athyrium brevifrons* Nakai ex Kitagawa	*Carex forficula* Franch. et Sav., *Linnaea borealis* Linn., *Pyrola incarnata* Fisch. ex DC.	*Mitella nuda* Linn., *Deyeuxia langsdorffii* (Link) Kunth, *Viola hamiltoniana* D.Don, *Oxalis corniculata* Linn., *Carex pilosa* Scop.	*Deyeuxia langsdorffii* (Link) Kunth, *Linnaea borealis* Linn.	*Deyeuxia langsdorffii* (Link) Kunth

MAT, mean annual temperature; MAP, mean annual precipitation; SAN, available soil nitrogen; SAP, available soil phosphorus; STN, total soil nitrogen; STP, total soil phosphorus.

### Root and soil sampling

We established six sampling sites (A–F) with an elevational gradient along the northern slope of Changbai Mountain: 540, 753, 1286, 1812, 2008, and 2357 m (**Supplementary Figure S1**). A stratified survey was used to investigate the community structure of the forests. Four primary quadrats (30 × 40 m), one or two nested sub-quadrats (5 × 5 m), and four nested sub-quadrats (1 × 1 m) were established in representative communities of each forest site for the survey of the tree, shrub, and herb layers, respectively (the quadrat numbers of each site are shown in **Table 1**). Species number, plant height, breast height (DBH) diameter, and species biomass of all trees with DBH ≥ 2 cm, all shrubs with basal stem diameter ≥ 2 cm, and all herbs in quadrats were measured. The climatic variables, including MAT and MAP for each sampling site, were derived from Chi et al. and Zhu et al.

Plant and soil samples were collected from six sites along an elevational gradient. Whole mature individual plants were collected for small herb species, and roots less than 2 mm were screened and transported to the laboratory for post-processing. To sample the roots of the trees and shrubs, we first loosened the soil within 2 m of the stem of the target plant on one side. Then, the root branches were followed by the tree stem to confirm the plant species. Subsequently, roots less than 2 mm in size were cut from the main lateral woody roots. The root samples were carefully washed to remove soil and other materials, oven-dried at 60°C in the laboratory and then ground in a ball mill for chemical analysis. Three replicates were performed for each species to ensure data reliability. A total of 612 individuals were collected from 204 species, with three replicates per species.

At each site, the soil was randomly sampled from 30 to 50 points in the 0–10 cm and 10–30 cm layers, and soil samples from the same depth were mixed. The soil samples were screened (with 2 mm mesh), air-dried in the ventilation chamber, and then ground in a ball mill for chemical analysis. Thirteen chemical elements (C, N, K, Ca, Mg, S, P, Al, Fe, Na, Mn, Zn, and Cu), soil pH, available soil phosphorus, and soil available nitrogen were measured in mixed soil samples. The measured soil chemical elements and conventional indexes were used to explore the diversity of root chemical traits.

### Elemental analysis

Thirteen elements (C, N, K, Ca, Mg, S, P, Al, Fe, Na, Mn, Zn, and Cu) were analyzed in the root and soil samples. The elements that account for more than 1/10000 of the total weight of organisms are macro-elements (C, N, K, Ca, Mg, S, and P), and the rest are microelements (Al, Fe, Na, Mn, Zn, and Cu). C and N were measured using an element analyzer (Vario Max CN Element Analyzer; Elementary Hanau, Germany). A microwave grad system (Mars X Press Microwave Grad System; CEM, Matthews, NC) was used to extract elemental ions from the samples after tracing metal-grade 68% HNO_3_ (for root samples) and HF (for soil samples) acidification. The elemental concentrations were analyzed using an inductively coupled plasma emission spectrometer (ICP-OES, Optima 5300 DV; PerkinElmer, Waltham, MA). Soil available N (SAN) was extracted with 2 mol L^-1^ KCL and measured using a continuous flow analyzer. Soil available P (SAP) was extracted from 5 g of air-dried soil with 100 ml 0.5 mol L^-1^ NaHCO_3_ and measured by the ammonium molybdate method.

### Statistical analysis

Root chemical traits were analyzed at both the species and community levels to explore the elevational patterns. At the species level, the coefficient of variation (CV) was used to evaluate the variability of fine root multiple elements concentrations (Zhao et al., 2016b).


$$CV=\frac{Standard\;Deviation}{Mean}$$

At the community level, the community-weighted mean (CWM) was used to evaluate the chemical element content in fine roots. The community-weighted variance (CWV) was used to evaluate the variability of multiple fine root elements (Hulshof et al., 2013). It should be noted that the collected species were divided into independent growth layers: tree, shrub, and herb layers, which were calculated separately at the community level. To do this, the CWM and CWV (Violle et al., 2007) of the elements were calculated for each plot j as follows:


$$CWM_{j}=\sum_{i=1}^{S}W_{ij}\times trait_{ij}$$

$$CWV=\sum_{i=1}^{S}W_{ij}\times {\left(trait_{ij}-CWM_{j}\right)}^{2}$$

where *W_ij_* is the relative abundance (diameter at breast height, DBH for trees; basal diameter for shrubs; aboveground biomass for herbs) of species *i* in plot *j*, and trait*_ij_* is the root element concentration of species *i* in plot *j*.

Linear regression was used to analyze the changes in root chemical concentrations and CWM along the elevation gradient (Zhu et al., 2010). One-way analysis of variance (ANOVA) and Duncan’s *post hoc* test was used to examine the differences in root chemical concentrations among the different PFT.

To explore the change of community chemical diversity along the elevation, and the influence of species diversity and environmental factors on community chemical diversity, we quantified the community chemical diversity of fine roots using the functional diversity Rao’s Q index (Rao Q). Rao (Rao, 1982) defined quadratic entropy as Rao Q based on species abundance in each quadrat and a measure of pairwise functional trait differences. We calculate Rao Q of the fine-root multi-element through the “dbFD” function of the “FD” package of R. Many distance functions have been developed to compare quantitative data (Botta-Dukat, 2005). Here, it is suggested to use the Euclidean distance to measure the difference in paired functional traits. Species diversity was calculated using the “vegan” package “diversity” function in R. Linear regression, and binomial regression analysis was used to analyze the changes in community chemical diversity and species diversity along the elevation gradient (Zhu et al., 2010). General linear regression was used to analyze the relationship between Rao Q and species diversity index.

Three species diversity indices were selected to evaluate the species diversity of tree, shrub, and herb layers in Changbai Mountain: species richness (S), Shannon index, and Simpson index (Gray, 2000; Keylock, 2005).


$$Shannon=-\sum_{i=1}^{S}P_{i}\;lnP_{i}$$

$$Simpson=1-\sum_{i=1}^{S}P_{i}^{2}$$

where *P_i_* is the relative importance value of species *i* and *S* is the total number of species in the quadrat of species *i*.

A general linear model (GLM) was used to explore the key factors that control community chemical assembly belowground. GLM can eliminate multi-collinearity between multiple independent variables, thereby increasing the reliability of the analysis (Heikkinen et al., 2005; Zhao et al., 2014). In this study, GLM was mainly applied in three aspects: (i) at the species level, the effects of PFT, climate, and soil on fine root chemical traits; (ii) at the community level, species diversity, climate, and soil effects on fine root chemical traits; and (iii) effects of species diversity, climate, and soil on fine root chemical diversity Rao Q. Detailed analysis steps were as follows: first, stepwise regressions were used to determine the climate (MAP and MAT) and soil variables (element concentration in soil; soil pH; soil available nitrogen [SAN], and soil available phosphorus [SAP]) to exclude variables that did not contribute significantly to the explained variation. Second, GLM was used to separate the variations into different fractions: (i) pure effect of species diversity, (ii) pure effect of climate, (iii) pure effect of soil, and combined variation due to the joint effects of (iv) species diversity and climate, (v) species diversity and soil, (vi) climate and soil, (vii) the three groups of explanatory variables, and (viii) unexplained variation. Logarithmic conversion of the 13 elements was performed to improve the normality of the data before analysis.

One-way ANOVA and Duncan’s *post hoc* test analysis was performed using SPSS. Linear and binomial regression analyses were performed in R using the “lm” package.

## Results

### Variations in root chemical traits at the species level

Root chemical traits exhibited extreme variation in the concentrations of 13 elements at the species level. The elemental concentrations ranged from 457.50 mg g^−1^ for the most abundant element C to 0.013 mg g^−1^ for the least abundant element Cu (**Supplementary Figure S2**). The concentrations of macronutrients (N, K, Ca, Mg, S, and P) varied from 12.20 mg g^−1^ (N) to 1.38 mg g^−1^ (P). In contrast, the concentrations of micronutrients (Fe, Na, Mn, Zn, and Cu) ranged from 0.76 mg g^−1^ (Fe) to 0.012 mg g^−1^ (Cu) (**Supplementary Figure S2**). The variability of the 13 elements differed widely with the CV increased from 0.07 for root C to 1.70 for root Cu (**Supplementary Table S1**).

Root chemical traits for the 13 elements showed significant variation among different PFT. Root C and Ca were remarkably higher in woody species than in herbaceous ones, whereas macronutrients (N, K, Mg, S, and P) were highest in herbs. There was no significant difference in the concentrations of trace metals (Fe, Mn, Zn, and Cu) between woody plants and herbs (**Supplementary Table S1**).

**Figure 1:**
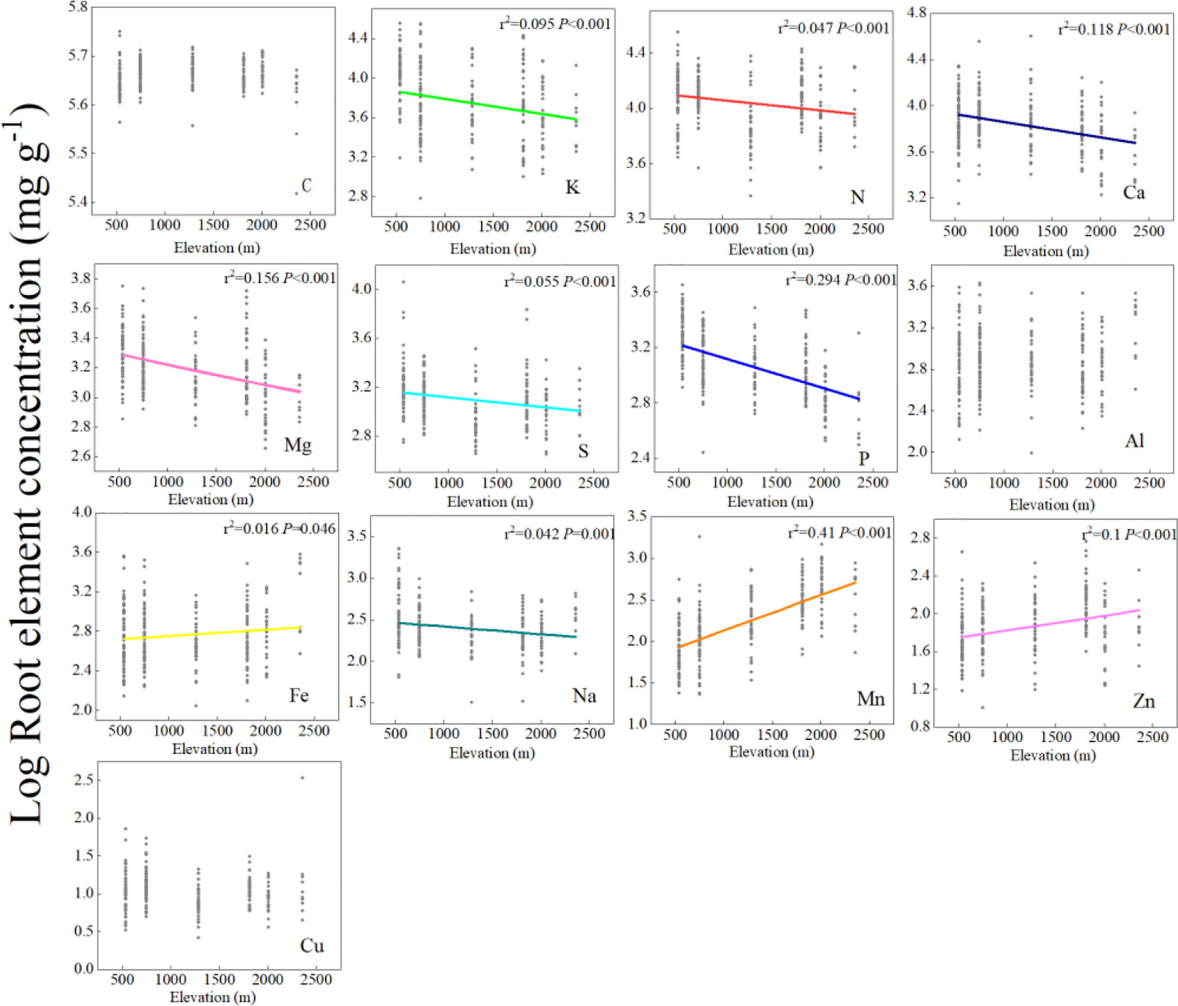
Linear regressions between fine root element concentrations and the elevation gradient at the species level. The *x*-axis represents elevation, the *y*-axis represents the Log concentrations of fine root elements, and each point in each mini-graph represents a species. Lines are plotted if regressions were significant at *P<* 0.05. A log scale is used on the *y*-axis.

Root chemical traits exhibited a significant elevation pattern. The concentrations of macronutrients (N, K, Ca, Mg, S, and P) decreased, whereas the trace metals (Fe, Mn, and Zn) increased with elevation (**Figure 1**). PFT, climate, and soil jointly influenced the elevation patterns of fine root chemical concentrations. GLM was used to determine the explanatory capacity of PFT, climate, and soil for different fine root chemical traits along the elevation gradient on Changbai Mountain (**Supplementary Table S2**). Our results showed that soil was the largest contributor to the explained variations in the fine root N, P, Mg, Mn, Zn, and Cu. PFT was the largest contributor to the explained variations in the fine root C, K, S, Al, and Na concentrations. Climate was the largest contributor to the explained variations in fine root Fe and Ca (**Supplementary Table S2**).

### Variations in root chemical traits at the community level

**Figure 2:**
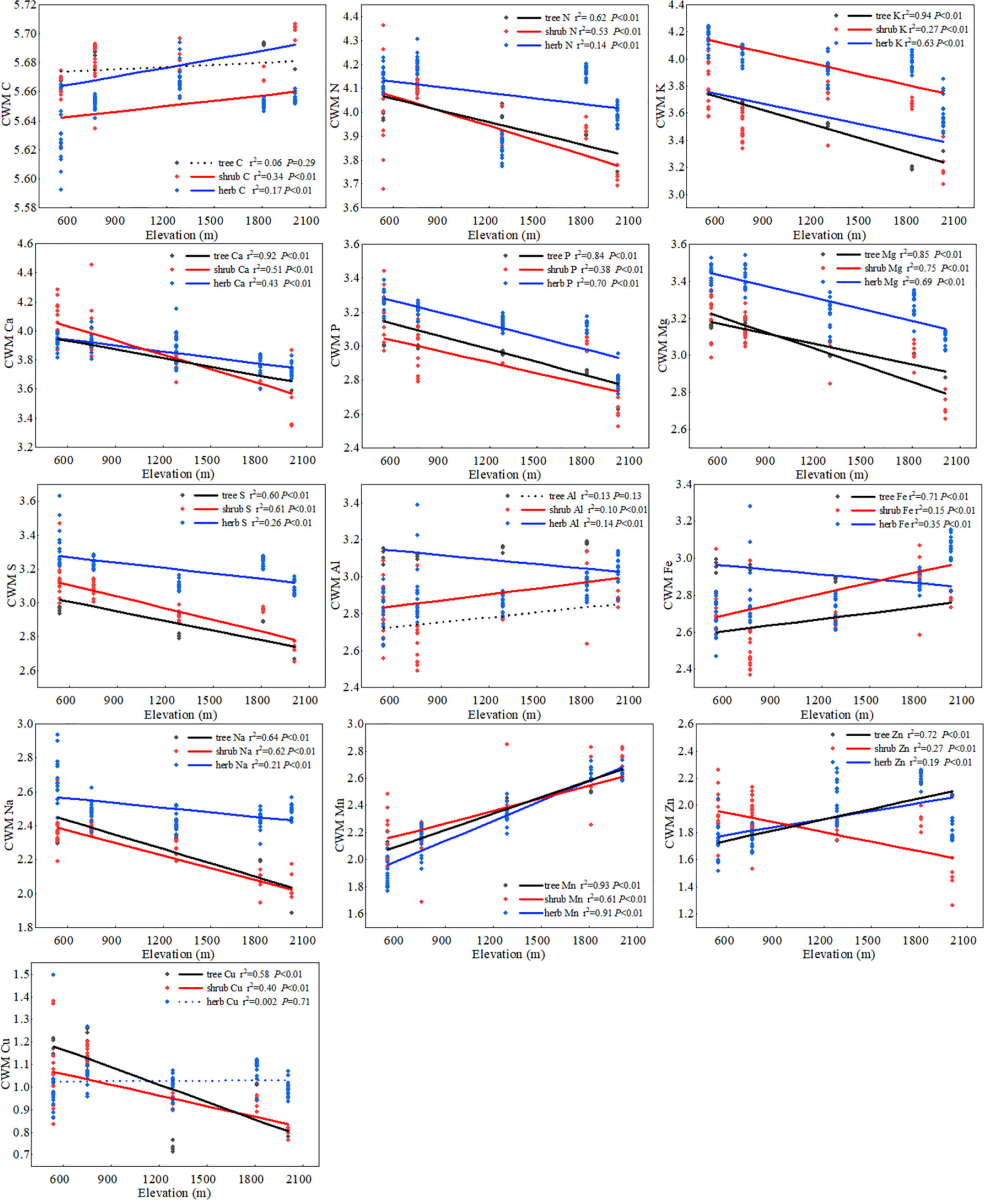
The elevational patterns of community root chemical traits. In each figure, black indicates the tree layer, red indicates the shrub layer, and blue indicates the herb layer. The *x*-axis represents the elevation gradient, and the *y*-axis represents the CWM value calculated by the Log of the elements. Solid lines are plotted if regressions were significant at *P*< 0.05; otherwise, a dashed line is plotted.

Root chemical traits at the community level exhibited significant differences among the five forest types along the elevation transect. The roots of BL and MCB were rich in N, K, Ca, Mg, S, P, Na, and Cu, whereas the CWMs of Al, Zn, and Mn were the highest in DCS and EB (**Supplementary Figure S3** and **Table S3**). Community chemical traits of root systematically shifted along elevational gradients and showed a pattern similar to that at the species level. The CWM of most fine root elements (N, K, Ca, Mg, S, P, Na, and Cu) decreased significantly from low to high elevations. In contrast, the CWM of trace metal elements (Fe, Mn, and Zn) showed an opposite trend in the herb, shrub, and tree layers (**Figure 2**; **Supplementary Table S4**). The CWV of macro-elements (N, K, Ca, P, Mg, and S) in the shrub and herb layers decreased significantly with increasing elevation, whereas CWV of metal elements (Fe and Mn) increased significantly with elevation. In the tree layers, CWV of P, Zn, and Fe increased significantly with elevation, whereas that of Cu decreased significantly with elevation. The CWV of the other elements showed no significant relationship with the elevation gradient (**Supplementary Figure S4**).

**Figure 3:**
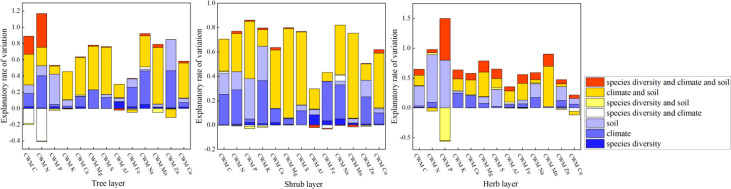
Summary of the General Linear Models (GLMs) for the effects of species diversity, climate, and soil on root chemical traits at the community level. Traits are the CWM of elements.

Fine root chemical traits at the community level are mainly influenced by abiotic factors (climate and soil) rather than biological factors (species diversity) (**Figure 3**; **Supplementary Table S5**). Community chemical traits in herbaceous and woody layers (tree and shrub layers) were affected by different environmental factors. In the herb layer, the CWM of most elements was affected by soil, except for K and Ca, which were affected by climate. In woody layers (tree and shrub layers), CWM of C, K, Ca, Mg, S, Al, Mn, and Cu are mainly regulated by the interactions of climate and soil, whereas P in the tree layer is mainly regulated by soil factors.

### Root chemical diversity variations

**Figure 4:**
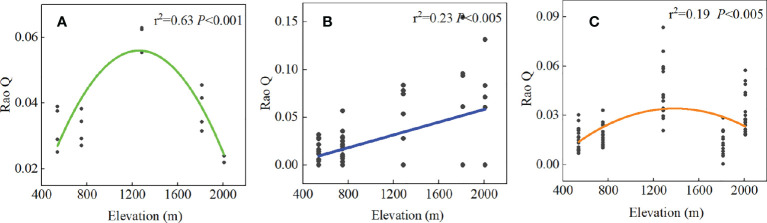
The relationship between the Rao Q of different PFTs fine root chemical traits and the elevation gradient. **(A)** denotes the tree layer, **(B)** denotes the shrub layer, and **(C)** denotes the herb layer.

**Figure 5:**
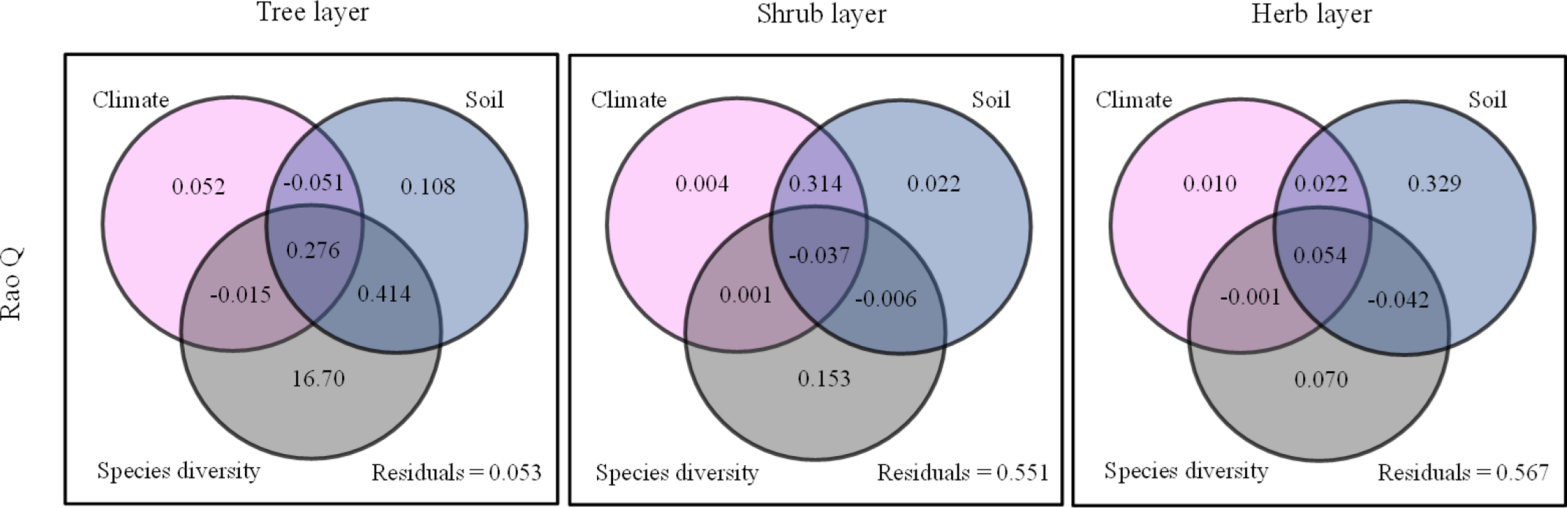
Summary of the General Linear Models (GLMs) for the effects of species diversity, climate, and soil factors on Rao Q. The gray circles denote species diversity factors; the pink circles denote climate factors; the blue circles denote soil factors.

Root chemical trait diversity showed significant elevational patterns (**Figure 4**). Rao Q differed significantly among the five forest types. With an increase in elevation, Rao Q of the shrub layer increased gradually and reached the maximum value at high elevations. Rao Q of the herb and tree layers first increased and then decreased, reaching a maximum value at middle elevations. Through GLM analysis, we found that Rao Q in the tree layer was mainly regulated by species diversity, Rao Q in the shrub layer was mainly regulated by the interactions of climate and soil, and Rao Q of fine roots in the herb layer was mainly regulated by soil factors (**Figure 5**; **Supplementary Table S7**).

## Discussion

### Multiple element concentrations in fine roots of different element types and different PFT

Root element contents affect the degree of variation in element concentrations. Our results showed that the variation in the macro-elements (C, N, K, Ca, Mg, S, and P) was smaller than that in the microelements (Al, Fe, Na, Mn, Zn, and Cu) (**Supplementary Table S1**). Previous studies exhibited that the CV increased from 7.0% root C to 87.4% for root Mn (Zhao et al., 2016a), and the concentrations of macro-elements (C, N, K, Mg, and S) and trace metals in roots varied 1–2 times and 3–9 times, respectively (Zhao et al., 2018a). The variability of root elements was similar to that of plant leaves across China (Zhang et al., 2012; Hao et al., 2015). Studies have shown that macroelements are strongly conserved, whereas trace elements have wide variability (Han et al., 2011; Zhao et al., 2016a). The restriction element stability hypothesis explains that high concentrations and, often, restricted elements in nature are less sensitive to environmental gradients (Han et al., 2011). In our study, the variation range of root elements along an elevational gradient (from 540 to 2357 m) (7–100%) was significantly smaller than the variability of elements along a latitude gradient (108.9° E, 18.7° N to 123.0° E, 51.8° N) (40–500% in Han et al., 2011, 35–120% in Zhao et al., 2016a) (Han et al., 2011; Zhao et al., 2016a). This difference suggests that the effect of the geographical scale must be considered when investigating element variability. The content of macroelements in nature is sufficient to supply plant demands fully, so there is no large variation due to environmental effects. However, because of the low content of trace elements, these are easily affected by the environment and the plant itself. Our study further confirms this phenomenon.

Plant root chemical traits showed great diversity among different PFTs. Woody plants are rich in structural elements (C and Ca) that form the cytoskeleton and cell walls. In contrast, herbaceous plants are rich in nutrients (N, K, Mg, Na, and S) related to plant growth, protein synthesis, and stomata (**Supplementary Table S1**) (Marschner, 2011). Wright et al. (2004) proposed the leaf economics spectrum (LES) concept. LES illustrates a universal LES consisting of key chemical, structural, and physiological properties (Wright et al., 2004). In our study, herbs had higher N, Mg, S, and Na concentrations, whereas trees had lower concentrations. This is because quick-investment return species (herbs) require more N, Mg, S, and Na to support rapid growth. Wen et al. (2021) showed that herbs had higher P, K, and Na and lower C:P and N:K ratios for different plant species than trees and shrubs (Wen et al., 2021). Herbaceous plants contain more nutrients because rapidly growing plants need sufficient nutrients to maximize their growth and development during the short span of the growing season (Kerkhoff et al., 2006; Zhang et al., 2018). Studies have consistently found that woody plants exhibit higher C concentrations than herbaceous plants. Conifers, relative to broad-leaved woody species, had higher C concentrations in their roots, leaves, and stems. The requirements for each essential nutrient are associated with the metabolic processes of the plants with different growth strategies (Sardans and Penuelas, 2014; Sardans et al., 2015; Penuelas et al., 2019; Sardans et al., 2021). The biogeochemical niche hypothesis states that each species has an optimal elemental composition and stoichiometry for optimal functioning to adopt a certain combination of metabolic and physiological processes (Penuelas et al., 2008; Urbina et al., 2017).

### Regulation of root chemical traits by PFT, climate, and soil

PFT, climate, and soil are the major factors shaping the complex biogeographic patterns of root chemical traits (Reich and Oleksyn, 2004; Yuan et al., 2011). Our study showed that CWM of root C concentration of shrub and herb layers increased gradually with elevation (**Figure 2**). This may be an ecological strategy for plants to cope with low temperatures (Zhao et al., 2018a). Non-structural carbon (including starch, low-molecular-weight sugars, and storage lipids) balances the osmotic pressure of cells, and plants at high elevations are subjected to more stress than those at low elevations. Therefore, higher concentrations of C accumulate to resist freezing (Hoch and Korner, 2012). Ecological researchers have proposed two hypotheses to explain the accumulation or deficiency of nutrients in plant tissues at high elevations (Oleksyn et al., 2002; Richardson, 2004). The temperature-plant physiology hypothesis indicates that plants maintain high nutrient concentrations when growing at high elevations to avoid cold injury and maintain metabolic capacity (Oleksyn et al., 2002). The temperature-biogeochemistry hypothesis states that temperature decreases with elevation, and soil microbial activity and plant metabolism decrease, thus limiting soil nutrient cycling and plant absorption (Kabata-Pendias, 2004; Thebault et al., 2014). In this study, the concentration of trace metals (Fe, Mn, and Zn) increased with elevation, which is consistent with the temperature-plant physiology hypothesis that plants maintain higher nutrient concentrations in their tissues to maintain metabolic capacity and avoid damage caused by low and high temperatures (**Figures 1** and **2**). Wang et al. (2018) observed the same phenomenon in the elevational distribution patterns of trace elements in plant–soil systems. At higher elevations, micronutrient concentrations were higher in roots of trees and shrubs to maintain physiological and ecological processes in colder environments (Wang et al., 2018), and the concentration of macroelements (N, K, Ca, Mg, S, and P) decreased significantly with elevation (**Figures 1** and **2**). This finding was consistent with the temperature-biogeochemistry hypothesis: Soil biological activity was reduced under low-temperature stress, resulting in slow decomposition of soil organic matter, limited mineral nutrient supply, and reduced absorption of elements by roots (Wright et al., 2004; Zhao et al., 2018b). The tendency of the macroelement (N, K, Ca, and Mg) concentration to decrease with elevation was also found in the fine roots of tropical montane forests (Graefe et al., 2010). In addition, increased precipitation in high-elevation areas led to increased soil leaching and decreased soil nutrient availability, which is also the cause of insufficient root element content in plants (Hedin et al., 2003).

Chemical elements are the basis of the plant body, and these chemical elements are relatively stable in the plant body. Herbaceous plants are short-lived plants with rapid growth and high income (Wright et al., 2004). Our results show that the community chemical traits of the herbaceous layer were mainly regulated by soil factors (**Figure 3**; **Supplementary Table S5**). Soil contains a large library of nutrients (Hou et al., 2020). The chemical composition of the fine roots of herbaceous plants is directly affected by changes in soil element concentrations. The rate and concentration of soil supply elements directly affect the growth of herbaceous plants (Hogan et al., 2021). Hogan et al. (2021) found that the concentrations of N, B, P, K, Mn, Cu, and Zn in root tissues increased with increasing soil nutrient availability. Pierick et al. (2021) found that topography and related soil fertility caused considerable small-scale variations in fine root traits and functional diversity in tropical montane forests (Pierick et al., 2021). Dominant species with strong competitiveness in the herbaceous layer can improve the internal stability of the plant body. However, the chemical traits of woody plants were mainly regulated by the interactions of climate and soil (**Figure 3**; **Supplementary Table S5**). Compared with herbaceous plants, woody plants are long-lived, and their fine root chemical traits change under the long-term influence of climate. The internal stability of woody plants was better than that of herbaceous plants. Biological homeostasis refers to an organism’s ability to maintain a relatively constant chemical composition in a changing environment (Kooijman, 1995). This hypothesis vividly reveals the relationship between the change in the environmental element ratio and the biological element ratio (Sterner and Elser, 2002).

### Chemical diversity and community chemical assembly

Species diversity and community chemical diversity showed significant elevation patterns. The environmental heterogeneity of the elevation gradient results in a variety of species diversity patterns. In our study, the species diversity of trees and herbs decreased significantly with elevation (**Supplementary Table S6**). The environmental energy hypothesis holds that the geographical pattern of species diversity is mainly caused by the direct energetic control of the physiological activities of species, and energy changes species diversity by directly affecting the physiological regulation mechanisms of individual organisms (Currie, 1991; Huang et al., 2017). Favorable environmental conditions allow more species to coexist, but temperatures and precipitation gradually decrease with elevation, and species that cannot adapt to extreme environments are lost. Mediterranean species declined with elevation because they were negatively affected by decreasing temperature (Di Biase et al., 2021).

Our study showed that the chemical diversity (Rao Q) of fine roots reached a maximum at middle elevations in the tree and herb layers (**Figure 4**). Rao’s quadratic entropy (Rao Q) represents the niche space overlap degree of a community. Higher Rao’s quadratic entropy leads to a weaker niche overlap degree of the species and weaker resource competition (Mason et al., 2005). In our study, Rao’s quadratic entropy of root element concentration of trees and herbs was the highest at middle elevations, indicating that plants at middle elevations utilize resources with high efficiency, improving ecosystem function (Diaz and Cabido, 2001). The chemical diversity of the shrub layer was highest at high elevations. Through GLM analysis, we found that Rao Q in the shrub layer was mainly regulated by the interactions between climate and soil. Therefore, the high Rao Q at high altitude may be related to the adaptation of shrubs to the environment and the utilization of resources. Rao Q has a positive effect on ecosystem processes, because highly diverse communities are better able to make optimal use of available resources, leading to increased ecosystem process rates. This mechanism has been termed the niche complementarity effect (Fox, 2005; Reiss et al., 2009; Searle and Chen, 2020). Based on the complementary effects, different species in the community have different ecological niches and chemical traits (Cardinale et al., 2007). Studies on chemical traits have shown that chemical diversity increases with the coexistence of species and species diversity (Fernandez-Martinez et al., 2021), whereas some studies showed that functional diversity remained unchanged or decreased with the increase of species diversity (Suárez-Castro et al., 2022). Many relationships existed between species diversity and functional diversity among PFTs (**Supplementary Figure 5**). In this study, the species diversity of the tree and herb layers significantly decreased with the increase of elevation, and the species diversity of the shrub layer also showed a decreasing trend, but not significantly (**Supplementary Table S6**), and the community structure tended to be simple at high elevations. The Rao Q of tree and shrub layers showed significant unimodal change with species diversity index (Richness, Simpson). The reason is that the species diversity of tree and shrub layers is higher and the content of chemical elements in the environment is more abundant at low elevation; however, due to the rich content of chemical elements, the requirements of each species for chemical elements are less limited, resulting in small differences in chemical traits among species. Therefore, Rao Q of tree and shrub layers at low elevation was small. In the middle elevation, the species diversity of tree and shrub layers is moderate, the ecological niche is well occupied, the environmental resources are fully utilized, the traits difference between species is large, and the functional diversity Rao Q is high. However, species diversity of tree and shrub layers decreased at high elevations, and the community structure tended to be simple at high elevations. Plants have evolved similar chemical traits to cope with low temperatures. Due to the convergence of plant functional traits, the complementarity of plant resource utilization decreases, leading to the decrease of chemical diversity. This is not only an adaptation to factors such as temperature, water, and species diversity at different elevations but also the result of environmental filtering (Mason et al., 2005). Hu et al. (2014) also found some correlation between species diversity and functional diversity in sandy plant communities (Hu et al., 2014).

The CWM describes the dominant functional trait value of the overall community by weighting species trait values based on species abundance (Lavorel et al., 2008). This can better reflect the mass ratio of species and community chemical assemblies (Lohbeck et al., 2015). This study showed that the weighted average value of the fine root chemical element community was mainly affected by the climate and soil rather than species diversity. Herb plants adopt a rapid growth strategy (Miller and Hawkins, 2003; Wright et al., 2004). According to the homeostasis hypothesis, strong species competitiveness can effectively maintain internal stability in plants. Dominant plants strongly influence ecosystem processes and control most of their resources (Hooper et al., 2005). This suggests that dominant plants may have high internal stability, because they control most resources. In contrast, rare plants have to take advantage of the resources left by the dominant plants. Most woody plants have slow growth and slow death strategy that affects the chemical composition of fine roots under long-term temperature and precipitation conditions (Agren and Weih, 2020). Climate is a major driver of the chemical construction of woody plant communities. However, the P content in woody plant fine roots is mainly regulated by soil factors because P is derived from mineral weathering in soil (Schreeg et al., 2013; Asner et al., 2014). Some metals, such as Al and Mn, are also regulated by soil factors in woody plants. Al and Mn are not essential elements in plants. However, many metal cations (Al^+3^, Mn^+2^) can be absorbed from acidic soil, resulting in excessive accumulation of Al and Mn in the soil and toxic effects on plants (Millaleo et al., 2010; Rehmus et al., 2017). Variations in chemical traits provide feedback on the function and organization of ecosystems and ultimately become another driver of life and environmental evolution.

## Conclusions

Our study improved the understanding of community chemical diversity of multiple elements and community chemical assembly belowground based on root chemical traits. The variation of root chemical traits at the species level was mainly regulated by PFTs and soil factors. Community chemical assembly of fine root were mainly driven by the interactions of climate and soil for woody layers, whereas soil factors played significant role for root chemical assembly for herb layer. The chemical assembly of rock-derived element P was mainly driven by soil factors. Meanwhile, root chemical diversities showed significant elevational patterns and were regulated by species diversity, the interactions of climate and soil, and soil factors in the tree, shrub and herb layers, respectively. However, this study is only a prelude to the root chemical traits and their significance in community chemical assembly. Further understanding of root morphological characteristics and structural diversity is required to fully understand the complex role of roots in community functional diversity and community assembly.

## Data availability statement

The raw data supporting the conclusions of this article will be made available by the authors, without undue reservation.

## Author contributions

SW performed the data analyses and wrote the manuscript; contributed to the conception of the study and contributed significantly to analysis and manuscript preparation. RW contributed to the experimental process and collect data. NZ contributed to the conception of the study; contributed significantly to analysis and manuscript preparation and helped perform the analysis with constructive discussions. HZ, YW, YD, SZ contributed to manuscript preparation. All authors contributed to the article and approved the submitted version.

## Funding

We thank for the support by the National Natural Science Foundation of China (No.32271619) and Gansu Province Science Foundation (No. 20JR10RA621).

## Conflict of interest

The authors declare that the research was conducted in the absence of any commercial or financial relationships that could be construed as a potential conflict of interest.

## Publisher’s note

All claims expressed in this article are solely those of the authors and do not necessarily represent those of their affiliated organizations, or those of the publisher, the editors and the reviewers. Any product that may be evaluated in this article, or claim that may be made by its manufacturer, is not guaranteed or endorsed by the publisher.
