# Supplementary material for: Changes in root chemical diversity along an elevation gradient of Changbai Mountain, China
Source: Front Plant Sci. 2022 Nov 7;13:897838. doi: 10.3389/fpls.2022.897838 (PMC9676470; doi:10.3389/fpls.2022.897838)
Supplement: Supplementary file 1 [file DataSheet_1.docx]

**Table S1.** The concentrations of multiple elements (C, N, K, Ca, Mg, S, P, Al, Mn, Fe, Na, Zn, Cu) in fine roots from Changbai Mountain. Differences among PFT were tested using one-way analysis of variance (ANOVA) and Duncan's post hoc test. Significant differences at P < 0.05 are indicated by different letters.

|  |  | C (mg g^-1^) | | N (mg g^-1^) | | K (mg g^-1^) | | Ca (mg g^-1^) | | P (mg g^-1^) | | Mg (mg g^-1^) | |
| --- | --- | --- | --- | --- | --- | --- | --- | --- | --- | --- | --- | --- | --- |
|  | n | mean | cv | mean | cv | mean | cv | mean | cv | mean | cv | mean | cv |
| All | 204 | 457.5 | 0.07 | 12.2 | 0.43 | 8.62 | 0.82 | 7.93 | 0.60 | 1.38 | 0.54 | 1.77 | 0.51 |
|  |  |  |  |  |  |  |  |  |  |  |  |  |  |
| tree | 36 | 481.35^a^ | 0.04 | 10.32^a^ | 0.43 | 3.84^a^ | 0.69 | 8.20^ab^ | 0.40 | 0.97^c^ | 0.38 | 1.33^a^ | 0.34 |
| shrub | 46 | 472.08^a^ | 0.06 | 11.10^a^ | 0.45 | 5.38^a^ | 0.64 | 9.37^b^ | 0.65 | 1.25^b^ | 0.57 | 1.44^a^ | 0.46 |
| herb | 122 | 442.52^b^ | 0.07 | 13.36^b^ | 0.40 | 11.7^b^ | 0.65 | 7.21^a^ | 0.61 | 1.59^a^ | 0.50 | 2.07^b^ | 0.49 |
|  |  |  |  |  |  |  |  |  |  |  |  |  |  |
| Broadleaf | 60 | 476.67^a^ | 0.05 | 11.46^a^ | 0.42 | 5.36^a^ | 0.62 | 9.84^a^ | 0.54 | 1.24^a^ | 0.51 | 1.52^a^ | 0.41 |
| Conifer | 4 | 470.06^a^ | 0.05 | 9.43^a^ | 0.80 | 2.99^b^ | 0.4 | 8.02^a^ | 0.57 | 0.83^b^ | 0.37 | 1.03^b^ | 0.24 |
|  |  |  |  |  |  |  |  |  |  |  |  |  |  |
| Evergreen | 6 | 488.16^a^ | 0.04 | 8.12^a^ | 0.84 | 2.62^a^ | 0.45 | 6.41^a^ | 0.75 | 0.77^a^ | 0.41 | 0.94^a^ | 0.29 |
| Deciduous | 59 | 474.27^a^ | 0.05 | 11.63^a^ | 0.41 | 5.42^b^ | 0.61 | 10.1^b^ | 0.52 | 1.25^b^ | 0.51 | 1.54^b^ | 0.39 |

|  |  | S (mg g^-1^) | | Al (mg g^-1^) | | Fe (mg g^-1^) | | Na (mg g^-1^) | | Mn (mg g^-1^) | | Zn (mg g^-1^) | | Cu (mg kg^-1^) | |
| --- | --- | --- | --- | --- | --- | --- | --- | --- | --- | --- | --- | --- | --- | --- | --- |
|  | n | mean | cv | mean | cv | mean | cv | mean | cv | mean | cv | mean | cv | mean | cv |
| All | 204 | 1.45 | 0.73 | 0.94 | 0.79 | 0.76 | 0.99 | 0.31 | 0.92 | 0.24 | 1.02 | 0.09 | 0.83 | 13.15 | 1.70 |
|  |  |  |  |  |  |  |  |  |  |  |  |  |  |  |  |
| tree | 36 | 0.95^a^ | 0.38 | 1.20^a^ | 0.53 | 0.79^a^ | 0.49 | 0.21^a^ | 0.5 | 0.20^a^ | 0.58 | 0.09^a^ | 0.66 | 12.69^a^ | 0.65 |
| shrub | 46 | 1.16^a^ | 0.45 | 0.90^b^ | 0.84 | 0.73^a^ | 0.98 | 0.25^a^ | 0.53 | 0.27^a^ | 0.94 | 0.09^a^ | 0.61 | 11.08^a^ | 0.4 |
| herb | 122 | 1.75^b^ | 0.72 | 0.86^b^ | 0.88 | 0.77^a^ | 1.13 | 0.38^b^ | 0.95 | 0.24^a^ | 1.13 | 0.09^a^ | 0.95 | 14.23^a^ | 2.07 |
|  |  |  |  |  |  |  |  |  |  |  |  |  |  |  |  |
| Broadleaf | 60 | 1.14^a^ | 0.44 | 1.01^a^ | 0.70 | 0.74^a^ | 0.74 | 0.25^a^ | 0.48 | 0.18^a^ | 0.83 | 0.08^a^ | 0.55 | 12.67^a^ | 0.52 |
| Conifer | 4 | 0.69^b^ | 0.22 | 1.42^a^ | 0.72 | 0.78^a^ | 0.51 | 0.20^a^ | 0.35 | 0.23^a^ | 0.62 | 0.07^a^ | 1.09 | 6.34^b^ | 0.42 |
|  |  |  |  |  |  |  |  |  |  |  |  |  |  |  |  |
| Evergreen | 6 | 0.69^a^ | 0.2 | 1.21^a^ | 0.78 | 0.76^a^ | 0.46 | 0.19^a^ | 0.29 | 0.38^a^ | 0.76 | 0.06^a^ | 1.13 | 6.71^a^ | 0.32 |
| Deciduous | 59 | 1.15^b^ | 0.43 | 1.04^a^ | 0.71 | 0.77^a^ | 0.79 | 0.25^a^ | 0.48 | 0.17^a^ | 0.68 | 0.09^a^ | 0.58 | 12.85^b^ | 0.52 |

**Table S2.** Summary of the General Linear Models (GLM) for the effects of plant functional types (PFT), climate, and soil on root chemical traits at the species level. a, b and c denote the independent effect of PFT, climate, and soil, respectively; ab, ac, and bc are respectively the interactive effect between PFT and climate, PFT and soil, climate and soil; abc denotes the interactive effect among the three factors.

|  | Total effects  (*R^2^*, %) | | | | Independent effects (*R^2^*, %) | | | Interactive effects  (*R^2^*, %) | | | |
| --- | --- | --- | --- | --- | --- | --- | --- | --- | --- | --- | --- |
|  | Full | PFT | Climate | Soil | a | b | c | ab | ac | bc | abc |
| Log C | 32.97 | 24.38 | 1.82 | 4.94 | 18.81 | 0.20 | 8.24 | 9.01 | 4.10 | 0.15 | -7.54 |
| Log N | 27.80 | 6.31 | 4.23 | 19.08 | 5.42 | 4.22 | 15.78 | -0.92 | 2.36 | 1.49 | -0.56 |
| Log K | 41.96 | 26.45 | 14.99 | 15.26 | 25.78 | 3.14 | 0.06 | -2.21 | 1.13 | 12.31 | 1.75 |
| Log Ca | 16.11 | 3.31 | 12.84 | 9.02 | 1.74 | 4.02 | 1.19 | 1.32 | 0.33 | 7.58 | -0.08 |
| Log Mg | 35.51 | 14.28 | 16.69 | 19.34 | 15.05 | 1.34 | 2.09 | 1.34 | 2.09 | 4.84 | -4.19 |
| Log S | 34.05 | 21.83 | 6.09 | 13.66 | 18.90 | 0.18 | 4.64 | 1.31 | 4.41 | 7.40 | -2.80 |
| Log P | 51.79 | 9.91 | 35.65 | 39.12 | 12.32 | 0.02 | 12.67 | 0.33 | -8.85 | 29.20 | 6.11 |
| Log Al | 9.99 | 6.35 | 1.66 | 0.37 | 6.14 | 2.47 | 2.16 | 1.01 | 0.03 | -0.99 | -0.83 |
| Log Fe | 13.96 | 2.05 | 2.57 | 0.05 | 3.71 | 11.83 | 9.25 | -1.63 | 6.73 | -9.17 | -6.76 |
| Log Na | 18.34 | 8.55 | 3.85 | 7.57 | 6.96 | 2.86 | 4.81 | 0.94 | 2.71 | 2.11 | -2.07 |
| Log Mn | 44.98 | 1.18 | 40.65 | 41.76 | 2.28 | 1.54 | 1.62 | -0.59 | 0.43 | 40.65 | -0.94 |
| Log Zn | 17.32 | 0.04 | 9.11 | 11.23 | 0.46 | 5.84 | 7.82 | -0.20 | -0.07 | 3.63 | -0.16 |
| Log Cu | 10.65 | 0.26 | 3.07 | 9.64 | 0.35 | 0.86 | 7.55 | -0.20 | -0.32 | 1.98 | 0.43 |

**Table S3.** The community weighted means of fine root multi-elements in the tree, shrub, and herb layers in different forest types. Differences among forest types were tested using one-way analysis of variance (ANOVA) and Duncan's post hoc test. Significant differences at P < 0.05 are indicated by different letters.

| Forest  layers | Community | n | CWM C (mg g^-1^) | CWM N (mg g^-1^) | CWM K (mg g^-1^) | CWM Ca (mg g^-1^) | CWM P (mg g^-1^) | CWM Mg  (mg g^-1^) |
| --- | --- | --- | --- | --- | --- | --- | --- | --- |
| tree layer | Broad leaved forest (BL) | 4 | 465.32^a^ | 9.49^a^ | 5.53^a^ | 8.55^a^ | 1.01^a^ | 1.43^a^ |
|  | Mixed coniferous broad leaved forest (MCB) | 4 | 487.52^b^ | 12.43^b^ | 4.56^b^ | 7.94^b^ | 0.99^a^ | 1.55^b^ |
|  | Dark coniferous spruce fir forest (DCF) | 4 | 460.67^c^ | 10.13^a^ | 3.23^c^ | 6.40^c^ | 0.91^b^ | 1.00^c^ |
|  | Dark coniferous spruce forest (DCS) | 4 | 492.95^d^ | 8.02^c^ | 1.56^d^ | 5.77^d^ | 0.70^c^ | 1.01^c^ |
|  | Ermans birch forest (EB) | 4 | 473.45^e^ | 5.64^d^ | 2.07^e^ | 3.85^e^ | 0.42^d^ | 0.76^d^ |
| shrub layer | Broad leaved forest (BL) | 20 | 453.62^a^ | 11.04^a^ | 6.63^a^ | 11.30^a^ | 1.44^a^ | 1.67^a^ |
|  | Mixed coniferous broad-leaved forest (MCB) | 16 | 479.96^c^ | 13.36^b^ | 3.65^b^ | 10.27^b^ | 1.11^b^ | 1.40^b^ |
|  | Dark coniferous spruce fir forest (DCF) | 12 | 480.59^c^ | 8.02^c^ | 5.81^ca^ | 6.54^c^ | 1.23^ba^ | 1.18^c^ |
|  | Dark coniferous spruce forest (DCS) | 17 | 465.39^b^ | 8.08^c^ | 4.92^bc^ | 5.96^c^ | 1.17^b^ | 0.91^d^ |
|  | Ermans birch forest (EB) | 24 | 504.35^d^ | 5.27^d^ | 1.70^d^ | 3.58^d^ | 0.41^c^ | 0.53^e^ |
| herb layer | Broad leaved forest (BL) | 16 | 425.05^a^ | 14.0a3^a^ | 14.37^a^ | 8.31^a^ | 1.84^a^ | 2.78^a^ |
|  | Mixed coniferous broad leaved forest (MCB) | 16 | 448.36^b^ | 16.19^b^ | 11.10^b^ | 8.61^a^ | 1.63^b^ | 2.69^a^ |
|  | Dark coniferous spruce fir forest (DCF) | 16 | 467.16^c^ | 7.25^c^ | 8.73^c^ | 8.04^a^ | 1.39^c^ | 1.73^b^ |
|  | Dark coniferous spruce forest (DCS) | 16 | 448.21^b^ | 14.89^a^ | 9.57^c^ | 5.94^b^ | 1.34^c^ | 1.93^b^ |
|  | Ermans birch forest (EB) | 16 | 451.95^b^ | 9.86^d^ | 4.14^d^ | 5.35^c^ | 0.62^d^ | 1.23^c^ |

| Forest layers | Community | n | CWM S (mg g^-1^) | CWM Al (mg g^-1^) | CWM Fe (mg g^-1^) | CWM Na (mg g^-1^) | CWM Mn (mg g^-1^) | CWM Zn (mg g^-1^) | CWM Cu  (mg kg^-1^) |
| --- | --- | --- | --- | --- | --- | --- | --- | --- | --- |
| tree layer | Broad leaved forest (BL) | 4 | 0.91^a^ | 1.30^ab^ | 0.91^a^ | 0.20^a^ | 0.15^a^ | 0.06^a^ | 15.35^a^ |
|  | Mixed coniferous broad leaved forest (MCB) | 4 | 1.18^b^ | 1.28^b^ | 0.91^a^ | 0.30^b^ | 0.16^b^ | 0.07^b^ | 17.47^b^ |
|  | Dark coniferous spruce fir forest (DCF) | 4 | 0.64^c^ | 1.42^c^ | 0.77^b^ | 0.22^a^ | 0.27^c^ | 0.06^a^ | 5.43^c^ |
|  | Dark coniferous spruce forest (DCS) | 4 | 0.78^d^ | 1.52^d^ | 0.81^b^ | 0.16^c^ | 0.31^d^ | 0.15^d^ | 10.29^d^ |
|  | Ermans birch forest (EB) | 4 | 0.46^e^ | 0.76^e^ | 0.66^c^ | 0.08^d^ | 0.43^e^ | 0.12^c^ | 6.01^c^ |
| shrub layer | Broad leaved forest (BL) | 20 | 1.32^a^ | 0.62^ab^ | 0.50^a^ | 0.23^a^ | 0.14^a^ | 0.08^a^ | 10.81^a^ |
|  | Mixed coniferous broad-leaved forest (MCB) | 16 | 1.23^a^ | 0.52^a^ | 0.35^b^ | 0.24^a^ | 0.14^a^ | 0.09^a^ | 13.66^b^ |
|  | Dark coniferous spruce fir forest (DCF) | 12 | 0.82^b^ | 0.61^ab^ | 0.45^ab^ | 0.19^b^ | 0.26^b^ | 0.06^b^ | 8.43^c^ |
|  | Dark coniferous spruce forest (DCS) | 17 | 0.89^b^ | 0.70^ab^ | 0.59^b^ | 0.10^c^ | 0.30^b^ | 0.09^a^ | 8.41^c^ |
|  | Ermans birch forest (EB) | 24 | 0.52^c^ | 0.76^b^ | 0.60^b^ | 0.11^c^ | 0.59^c^ | 0.03^c^ | 6.26^c^ |
| herb layer | Broad leaved forest (BL) | 16 | 2.18^a^ | 0.67^a^ | 0.49^a^ | 0.48^a^ | 0.08^a^ | 0.06^a^ | 10.60^ab^ |
|  | Mixed coniferous broad leaved forest (MCB) | 16 | 1.75^b^ | 0.90^ab^ | 0.68^bc^ | 0.31^b^ | 0.14^b^ | 0.05^a^ | 11.74^ab^ |
|  | Dark coniferous spruce fir forest (DCF) | 16 | 1.23^c^ | 0.72^a^ | 0.53^ab^ | 0.26^b^ | 0.22^c^ | 0.13^b^ | 10.17^ab^ |
|  | Dark coniferous spruce forest (DCS) | 16 | 1.76^b^ | 0.87^a^ | 0.75^c^ | 0.28^b^ | 0.43^d^ | 0.16^c^ | 12.22^b^ |
|  | Ermans birch forest (EB) | 16 | 1.22^c^ | 1.10^b^ | 1.15^d^ | 0.31^b^ | 0.43^d^ | 0.07^a^ | 9.77^a^ |

**Table S4.** Linear regressions of the community-weighted means (CWM) for fine root elements versus elevation for the tree, shrub, and herb layers. “***” denotes *P* < 0.001, “*” denotes *P* < 0.05.

|  | Elements | n | Slope | r^2^ | *P* |
| --- | --- | --- | --- | --- | --- |
| Tree layer | CWM C | 20 | 5.430 | 0.061 | 0.293 |
|  | CWM N | 20 | -3.130 | 0.620 | <0.001^***^ |
|  | CWM K | 20 | -2.525 | 0.938 | <0.001^***^ |
|  | CWM Ca | 20 | -2.805 | 0.920 | <0.001^***^ |
|  | CWM P | 20 | -0.351 | 0.837 | <0.001^***^ |
|  | CWM Mg | 20 | -0.475 | 0.849 | <0.001^***^ |
|  | CWM S | 20 | -0.329 | 0.596 | <0.001^***^ |
|  | CWM Al | 20 | -0.166 | 0.125 | 0.126 |
|  | CWM Fe | 20 | -0.147 | 0.708 | <0.001^***^ |
|  | CWM Na | 20 | -0.104 | 0.637 | <0.001^***^ |
|  | CWM Mn | 20 | 0.177 | 0.925 | <0.001^***^ |
|  | CWM Zn | 20 | 0.054 | 0.715 | <0.001^***^ |
|  | CWM Cu | 20 | -0.007 | 0.584 | <0.001^***^ |
| Shrub layer | CWM C | 89 | 21.100 | 0.336 | <0.001^***^ |
|  | CWM N | 89 | -4.276 | 0.530 | <0.001^***^ |
|  | CWM K | 89 | -2.102 | 0.273 | <0.001^***^ |
|  | CWM Ca | 89 | -4.932 | 0.509 | <0.001^***^ |
|  | CWM P | 89 | -0.497 | 0.384 | <0.001^***^ |
|  | CWM Mg | 89 | -0.693 | 0.749 | <0.001^***^ |
|  | CWM S | 89 | -0.484 | 0.609 | <0.001^***^ |
|  | CWM Al | 89 | 0.119 | 0.102 | 0.00234^**^ |
|  | CWM Fe | 89 | 0.117 | 0.153 | <0.001^***^ |
|  | CWM Na | 89 | -0.096 | 0.617 | <0.001^***^ |
|  | CWM Mn | 89 | 0.266 | 0.614 | <0.001^***^ |
|  | CWM Zn | 89 | -0.030 | 0.272 | <0.001^***^ |
|  | CWM Cu | 89 | -0.004 | 0.403 | <0.001^***^ |
| Herb layer | CWM C | 80 | 12.200 | 0.172 | <0.001^***^ |
|  | CWM N | 80 | -2.291 | 0.139 | <0.001^***^ |
|  | CWM K | 80 | -5.077 | 0.629 | <0.001^***^ |
|  | CWM Ca | 80 | -2.184 | 0.473 | <0.001^***^ |
|  | CWM P | 80 | -0.639 | 0.698 | <0.001^***^ |
|  | CWM Mg | 80 | -0.942 | 0.689 | <0.001^***^ |
|  | CWM S | 80 | -0.429 | 0.256 | <0.001^***^ |
|  | CWM Al | 80 | 0.186 | 0.139 | <0.001^***^ |
|  | CWM Fe | 80 | 0.319 | 0.354 | <0.001^***^ |
|  | CWM Na | 80 | -0.088 | 0.210 | <0.001^***^ |
|  | CWM Mn | 80 | 0.248 | 0.913 | <0.001^***^ |
|  | CWM Zn | 80 | 0.041 | 0.191 | <0.001^***^ |
|  | CWM Cu | 80 | -0.0002 | 0.002 | 0.710 |

**Table S5.** Summary of the General Linear Models (GLM) for the effects of species diversity, climate, and soil on root chemical traits at the community level. Traits are the community-weighted mean of elements. a, b and c denote the independent effects of species diversity, climate, and soil, respectively; ab is the interactive effect between species diversity and climate; ac is the interactive effect between species diversity and soil; bc is the interactive effect between climate and soil; abc is the interaction among species diversity, climate, and soil factors.

|  |  | Total effects (*R^2^, %*) | | |  | |  | | Independent effects (*R^2^, %*) | | | | Interactive effects (*R^2^, %*) | | | | |
| --- | --- | --- | --- | --- | --- | --- | --- | --- | --- | --- | --- | --- | --- | --- | --- | --- | --- |
|  | Traits | Full | Species diversity | Climate | | Soil | | a | | b | c | ab | | ac | bc | abc |  |
| Tree layer | CWM C | 69.20 | 4.47 | 58.05 | | 51.09 | | 1.98 | | 16.45 | 10.41 | -18.43 | | -1.24 | 37.86 | 22.16 |  |
|  | CWM N | 76.11 | 1.55 | 63.68 | | 47.89 | | 0.57 | | 39.63 | 12.41 | -40.19 | | -0.55 | 22.51 | 41.73 |  |
|  | CWM P | 83.14 | 0.00 | 45.70 | | 79.35 | | 1.90 | | 2.93 | 37.22 | -1.04 | | -1.69 | 10.13 | 0.83 |  |
|  | CWM K | 44.62 | 0.40 | 35.87 | | 42.19 | | 0.29 | | 2.35 | 7.56 | -0.21 | | 0.90 | 34.31 | -0.58 |  |
|  | CWM Ca | 63.52 | 4.07 | 61.02 | | 47.64 | | 1.90 | | 12.88 | 0.46 | 1.10 | | 0.15 | 46.11 | 0.92 |  |
|  | CWM Mg | 77.91 | 1.54 | 77.73 | | 54.92 | | 0.17 | | 22.75 | 0.00 | 0.07 | | 0.00 | 53.62 | 1.30 |  |
|  | CWM S | 75.97 | 1.06 | 72.26 | | 62.22 | | 0.11 | | 13.49 | 3.52 | 0.16 | | 0.08 | 57.91 | 0.71 |  |
|  | CWM Al | 27.42 | 6.66 | 14.46 | | 18.97 | | 8.27 | | 0.05 | 4.25 | 0.13 | | 0.44 | 16.47 | -2.18 |  |
|  | CWM Fe | 31.92 | 0.29 | 20.89 | | 6.80 | | 1.82 | | 24.29 | 10.24 | -0.99 | | -1.04 | -2.90 | 0.50 |  |
|  | CWM Na | 81.77 | 10.32 | 74.89 | | 33.02 | | 4.70 | | 41.25 | 2.37 | 2.80 | | -0.19 | 38.15 | 3.00 |  |
|  | CWM Mn | 73.83 | 0.48 | 68.57 | | 71.97 | | 1.50 | | 0.13 | 3.51 | 0.24 | | -5.01 | 69.71 | 3.75 |  |
|  | CWM Zn | 73.56 | 0.03 | 35.50 | | 27.36 | | 0.82 | | 45.74 | 37.90 | -0.36 | | -0.66 | -10.10 | 0.23 |  |
|  | CWM Cu | 58.21 | 4.47 | 51.04 | | 51.09 | | 1.55 | | 5.65 | 5.07 | -0.08 | | 0.54 | 43.02 | 2.45 |  |
| Shrub layer | CWM C | 70.37 | 1.89 | 52.59 | | 43.82 | | 0.00 | | 25.18 | 17.08 | 1.37 | | 0.70 | 26.23 | -0.19 |  |
|  | CWM N | 76.11 | 1.55 | 60.95 | | 47.89 | | 0.57 | | 28.20 | 14.90 | -0.54 | | -0.30 | 31.46 | 1.84 |  |
|  | CWM P | 83.14 | 0.00 | 49.89 | | 79.35 | | 1.90 | | 2.93 | 33.25 | -1.04 | | -1.90 | 46.96 | 1.04 |  |
|  | CWM K | 77.66 | 0.40 | 49.49 | | 42.19 | | 1.06 | | 35.39 | 28.13 | -0.98 | | -1.02 | 13.74 | 1.34 |  |
|  | CWM Ca | 63.62 | 4.07 | 61.02 | | 50.54 | | 1.99 | | 11.12 | 0.56 | -0.03 | | 0.05 | 47.88 | 2.05 |  |
|  | CWM Mg | 79.74 | 1.54 | 79.18 | | 73.95 | | 0.42 | | 4.79 | 0.24 | 0.58 | | -0.09 | 73.18 | 0.63 |  |
|  | CWM S | 76.76 | 1.06 | 72.24 | | 64.59 | | 0.04 | | 11.66 | 4.37 | 0.47 | | 0.11 | 59.67 | 0.44 |  |
|  | CWM Al | 27.42 | 6.66 | 14.46 | | 18.97 | | 8.27 | | 0.05 | 4.25 | 0.13 | | 0.44 | 16.47 | -2.18 |  |
|  | CWM Fe | 39.93 | 0.29 | 36.30 | | 6.80 | | 3.47 | | 32.30 | 0.12 | -2.64 | | 0.04 | 7.23 | -0.58 |  |
|  | CWM Na | 81.58 | 10.32 | 72.55 | | 43.88 | | 5.05 | | 27.81 | 3.17 | 4.85 | | 0.81 | 40.29 | -0.39 |  |
|  | CWM Mn | 73.83 | 0.48 | 68.57 | | 71.97 | | 1.50 | | 0.13 | 3.51 | 0.24 | | 0.25 | 69.71 | -1.51 |  |
|  | CWM Zn | 49.43 | 0.03 | 35.62 | | 26.89 | | 0.74 | | 22.26 | 13.73 | -0.46 | | -0.65 | 13.41 | 0.40 |  |
|  | CWM Cu | 61.88 | 4.47 | 58.05 | | 51.09 | | 0.64 | | 9.32 | 3.09 | 0.84 | | 0.10 | 45.00 | 2.90 |  |
| Herb layer | CWM C | 64.42 | 10.93 | 29.99 | | 61.55 | | 0.39 | | 2.64 | 33.44 | -0.15 | | 0.61 | 17.42 | 10.08 |  |
|  | CWM N | 92.66 | 8.80 | 9.60 | | 83.59 | | 0.15 | | 9.07 | 80.02 | -0.15 | | 2.89 | -5.22 | 5.91 |  |
|  | CWM P | 93.52 | 14.98 | 68.49 | | 93.47 | | 0.04 | | 0.01 | 79.90 | 0.00 | | -54.90 | -1.37 | 69.84 |  |
|  | CWM K | 63.41 | 16.92 | 60.48 | | 37.33 | | 0.00 | | 24.34 | 2.67 | 1.74 | | 0.26 | 19.48 | 14.92 |  |
|  | CWM Ca | 57.96 | 11.36 | 56.77 | | 37.38 | | 0.04 | | 20.47 | 1.19 | 0.07 | | -0.03 | 24.94 | 11.29 |  |
|  | CWM Mg | 78.67 | 19.06 | 67.52 | | 71.05 | | 0.02 | | 7.48 | 10.83 | 0.12 | | 0.29 | 41.29 | 18.63 |  |
|  | CWM S | 64.99 | 19.35 | 33.22 | | 62.44 | | 0.06 | | 2.43 | 28.57 | 0.07 | | 3.14 | 14.64 | 16.08 |  |
|  | CWM Al | 35.01 | 10.30 | 33.40 | | 26.72 | | 0.52 | | 4.76 | 1.43 | 3.01 | | -0.34 | 18.52 | 7.11 |  |
|  | CWM Fe | 55.05 | 14.87 | 48.33 | | 48.70 | | 0.76 | | 5.97 | 6.29 | -0.38 | | -0.33 | 27.93 | 14.81 |  |
|  | CWM Na | 59.04 | 13.03 | 34.73 | | 41.86 | | 0.01 | | 17.17 | 23.21 | 0.01 | | 1.10 | 5.64 | 11.91 |  |
|  | CWM Mn | 90.11 | 20.51 | 88.40 | | 89.53 | | 0.00 | | 0.54 | 1.70 | 0.04 | | 0.01 | 67.36 | 20.45 |  |
|  | CWM Zn | 44.24 | 5.77 | 20.76 | | 33.33 | | 1.54 | | 10.84 | 23.40 | -1.46 | | -1.45 | 4.23 | 7.15 |  |
|  | CWM Cu | 9.70 | 0.04 | 0.06 | | 9.45 | | 0.03 | | 6.04 | 9.63 | -5.82 | | -0.02 | -6.02 | 5.85 |  |

**Table S6.** Linear regression of species diversity (Shannon-Wiener index, Simpson index, and species richness index) on elevation.

|  |  | n | Slope | r^2^ | *P* |
| --- | --- | --- | --- | --- | --- |
| Tree layer | richness | 20 | -0.007 | 0.743 | <0.001 *** |
|  | shannon | 20 | -0.001 | 0.657 | <0.001*** |
|  | simpson | 20 | -0.0004 | 0.585 | <0.001*** |
| Shrub layer | richness | 89 | -0.0001 | 0.008 | 0.404 |
|  | shannon | 89 | -4.19E-05 | 0.004 | 0.547 |
|  | simpson | 89 | -2.11E-05 | 0.003 | 0.626 |
| Herb layer | richness | 80 | -0.003 | 0.258 | <0.001*** |
|  | shannon | 80 | -0.0002 | 0.137 | <0.001*** |
|  | simpson | 80 | -7.46E-05 | 0.106 | 0.00323** |

**Table S7.** Summary of the General Linear Models (GLM) for species diversity, climate, and soil effects on Rao Q index of root chemical traits. a, b, c denotes the independent effects of species diversity, climate, and soil, respectively; ab is the interactive effect between species diversity and climate; ac is the interactive effect between species diversity and soil; bc is the interactive effect between climate and soil; abc is the interaction of species diversity, climate, and soil.

|  | Total effects (*R^2^, %*) | | | | Independent effects (*R^2^, %*) | | | Interactive effects (*R^2^, %*) | | | |
| --- | --- | --- | --- | --- | --- | --- | --- | --- | --- | --- | --- |
|  | Full | Species diversity (a) | Climate (b) | Soil  (c) | a | b | c | ab | ac | bc | abc |
| Rao Q tree | 95.00 | 84.18 | 26.25 | 74.70 | 16.70 | 5.22 | 10.78 | -1.53 | 41.36 | -5.09 | 27.64 |
| Rao Q shrub | 45.00 | 11.09 | 28.21 | 29.27 | 15.27 | 0.40 | 2.16 | 0.11 | -0.59 | 31.39 | -3.69 |
| Rao Q herb | 43.30 | 7.21 | 7.62 | 36.30 | 7.00 | 0.96 | 32.91 | -0.96 | -4.23 | 2.23 | 5.39 |

**Figure S1.** Summary of vertical distribution of vegetation types in Changbai Mountain.

**
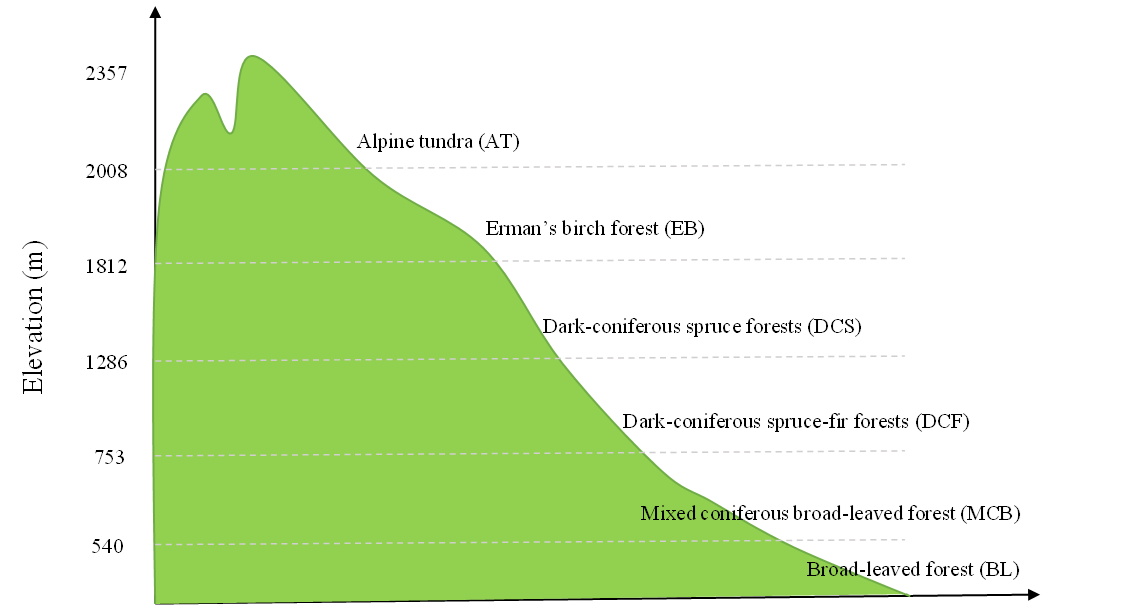
**

**Figure S2.** The average concentrations of 13 elements (C, N, K, Ca, Mg, S, P, Al, Mn, Fe, Na, Zn, and Cu) in the fine roots of 204 species collected from Changbai Mountain. (a) represents macro elements, and figure (b) refers to trace elements. The x-axis represents element classifications, and the y-axis represents element concentrations. A total of 204 species (n = 204). Columns and error bars represent the mean and standard error of element concentrations, with specific values on each column.


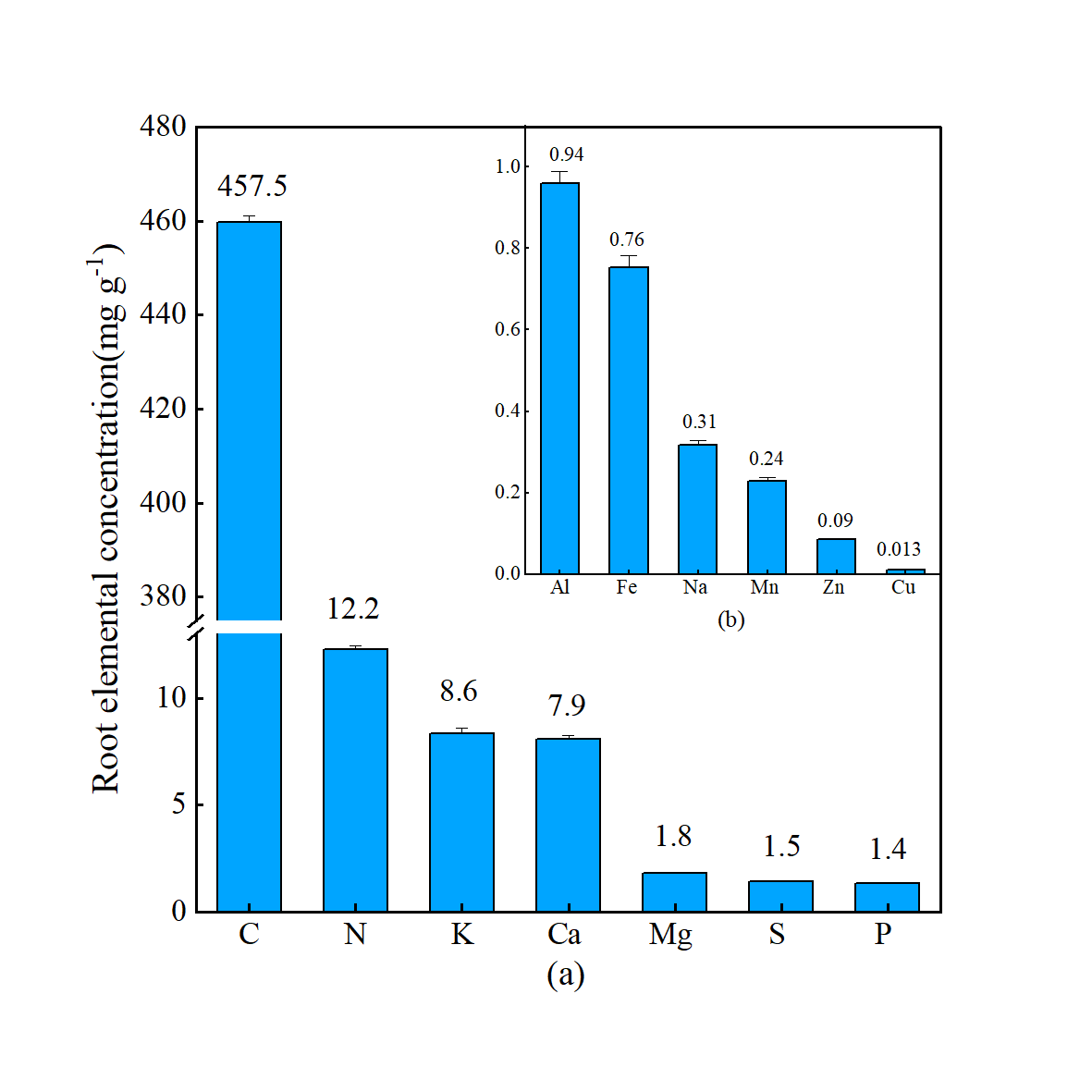


**Figure S3.** Community-weighted mean (CWM) values of the tree, shrub, and herb layer fine-root elements for each vegetation type. Orange represents broadleaf forests, green represents mixed coniferous broadleaf forests, purple represents dark-coniferous spruce-fir forest, yellow represents Erman’s birch forest, and blue represents alpine tundra. The x-axis represents the element classification, and the y-axis represents each element's community weighted mean values. Columns and error bars show the community weighted means and standard error. Different letters (a, b, and c) indicate significant differences at the *p* < 0.05 level.


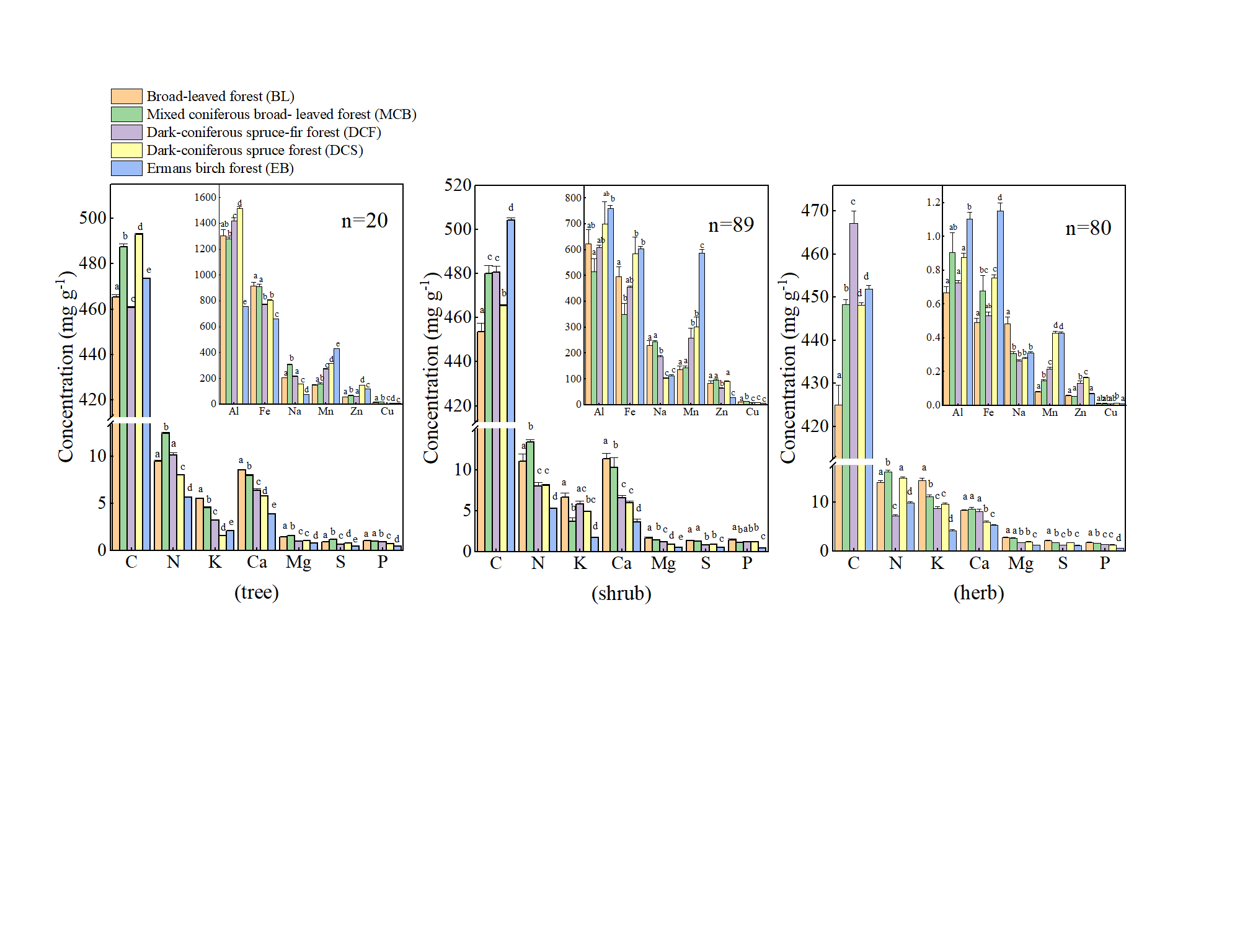


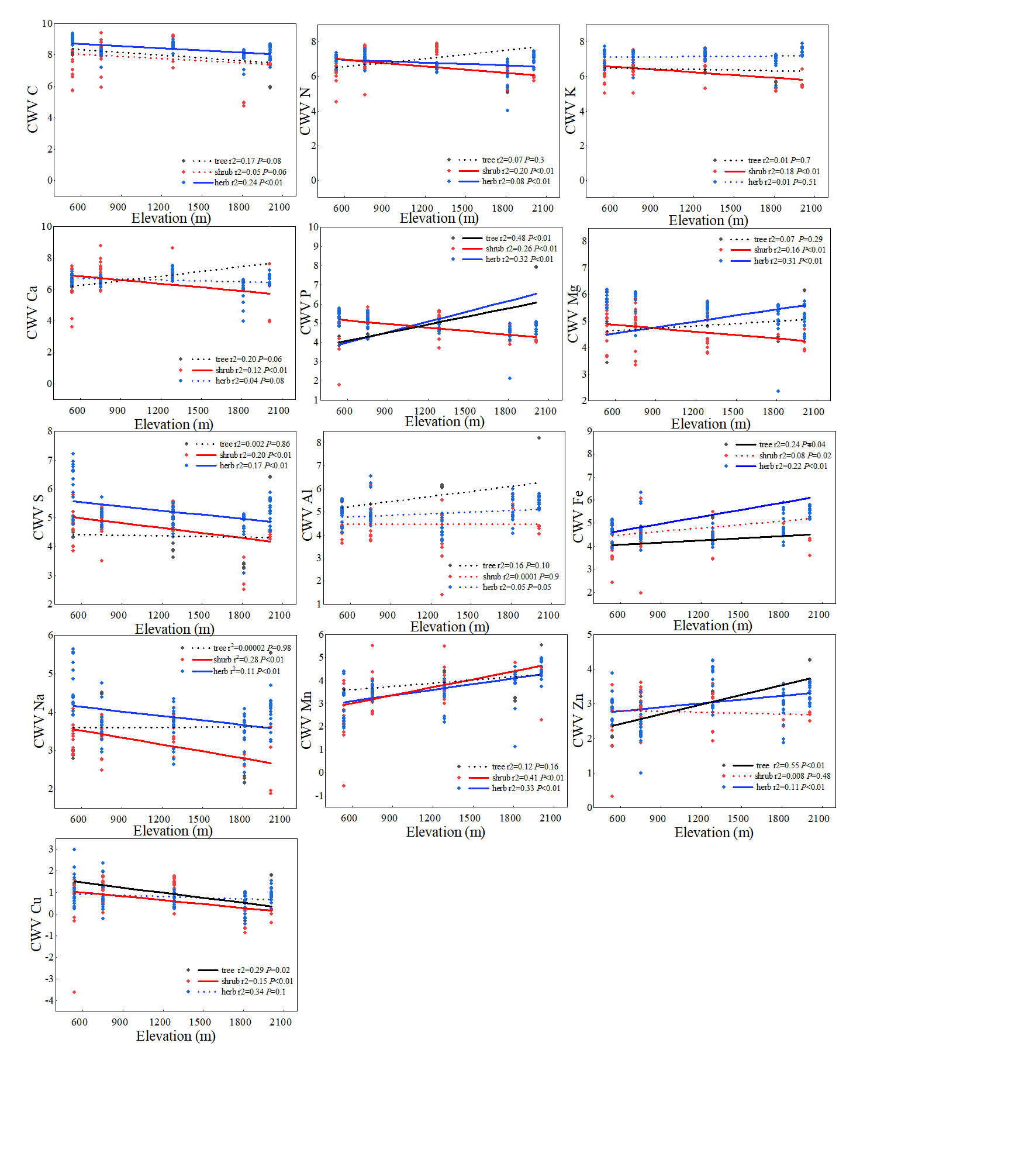
**Figure S4**. Linear regressions between community-weighted variance (CWV) of fine root chemical elements and the elevation gradient.


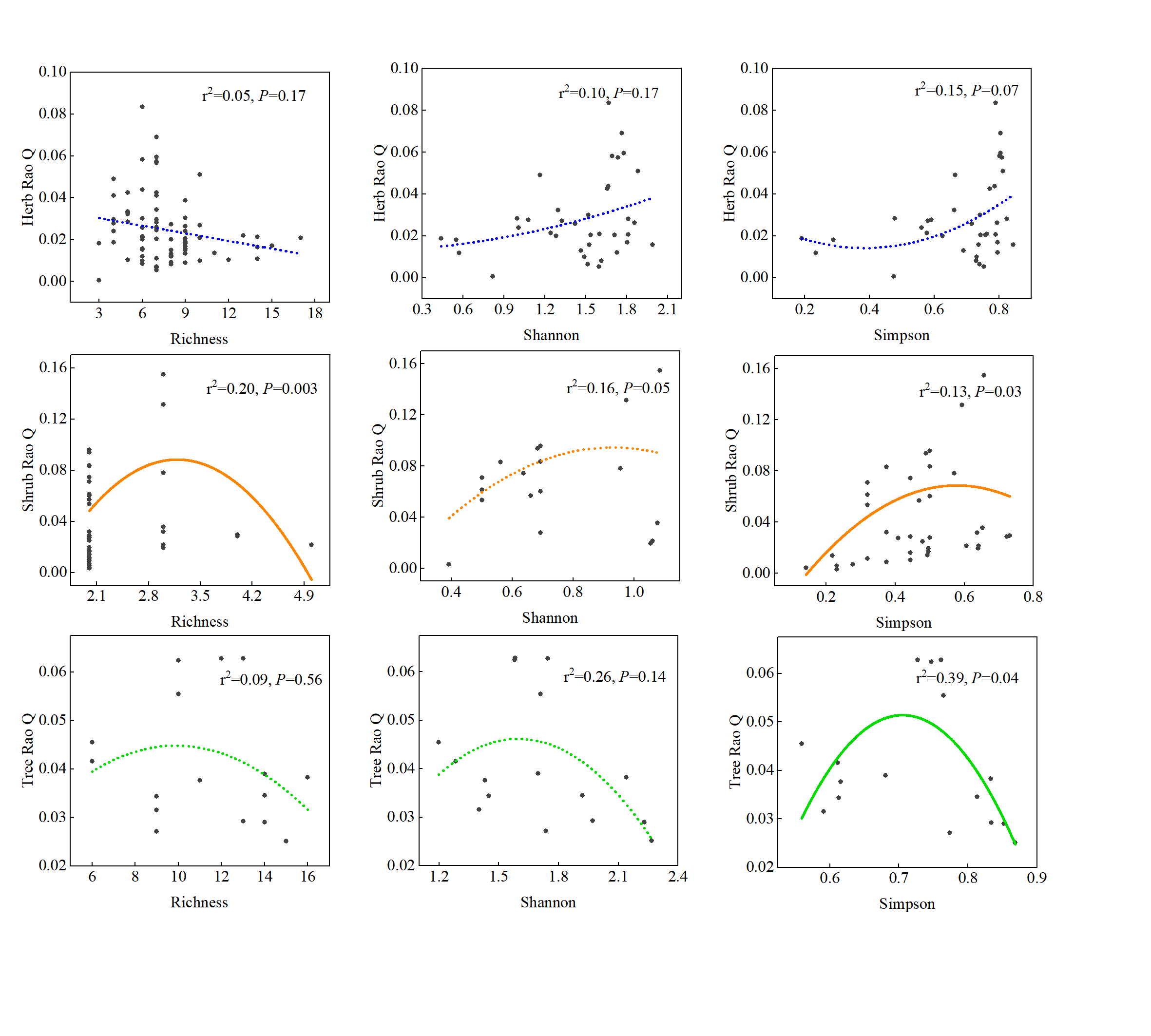
**Figure S5**. Relationship between Rao Q and species diversity index in tree, shrub and herb layers. Solid lines are plotted if regressions were significant at P<0.05; otherwise, a dashed line is plotted.
